# TET1 deficiency amplifies macrophage inflammatory signaling associated with Crohn’s disease

**DOI:** 10.1007/s00011-026-02228-3

**Published:** 2026-03-27

**Authors:** Rocio K. Perez, Reeba Paul, Parveen Kumar, Alp Tutkun, Deborah Webb, Shea McGorty, Thomas Wieckowski, Yoon Sing Yap, Tiffany E. Leesang, Panayiotis I. Vlantis, Aristeidis G. Telonis, Christine Hajdin, Jim King, Gerald Nabozny, Luisa Cimmino

**Affiliations:** 1 Immunology and Respiratory Department, Boehringer Ingelheim Pharmaceuticals, Inc, Ridgefield, CT USA; 2 Computational Biology and Digital Sciences Department, Boehringer Ingelheim Pharmaceuticals, Inc, Ridgefield, CT USA; 3 Department of Biochemistry and Molecular Biology, University of Miami, Miller School of Medicine, FL Miami, USA; 4 Sylvester Comprehensive Cancer Center, Miami, FL USA

**Keywords:** Crohn’s disease, TET1, Inflammatory macrophages, CCL-8, Neutrophil migration

## Abstract

**Objective and design:**

To define the role of Ten-Eleven Translocation (TET) proteins in Crohn’s disease (CD)-associated inflammation through integrative human and mechanistic studies.

**Material:**

Publicly available CD transcriptomic and DNA methylation datasets, and primary mononuclear cells and ileal biopsies were analyzed for TET gene expression and signatures. *TET1* and *TET2* CRISPR/Cas9 knockout macrophage cell lines were generated.

**Treatment:**

Macrophages were stimulated with LPS in the presence or absence of kinase inhibitors. Conditioned media from macrophages were applied to primary human neutrophils. PBMCs from CD patients and healthy donors were stimulated with LPS for validation.

**Methods:**

Macrophages or primary patients samples were analyzed by high-throughput surface marker profiling, RNA sequencing, 5hmC sequencing, assays of effector function, qRT-PCR, phosphoflow, and cytokine/chemokine release by ELISA.

**Results:**

*TET1* was the most downregulated TET enzyme in CD blood and ileal tissues, correlating with reduced TET-associated gene signatures and elevated inflammatory mediators. *TET1*-deficient macrophages exhibited distinct surface phenotypes, reduced *PTEN* expression, altered 5hmC distribution, and heightened IFN gene expression, ERK activation, and chemokine release associated with enhanced neutrophil migration. PBMCs from CD patients mirrored reduced *TET1* expression and exaggerated inflammatory responses.

**Conclusions:**

TET1 functions as a non-redundant regulator of inflammatory macrophages and aberrant chemokine signaling linked to immune cell recruitment in Crohn’s disease.

**Supplementary Information:**

The online version contains supplementary material available at 10.1007/s00011-026-02228-3.

## Introduction

Myeloid cells such as monocytes and macrophages are central players during inflammatory responses, regulating the recruitment of other immune and inflammatory cell types to address insult and injury, and driving resolution mechanisms to promote healing [1]. The differentiation of peripheral monocytes into macrophages can be driven by the response to inflammatory signals like those engaging innate immune signaling receptors (i.e. TLR, NOD 1/2) or through chemokine ligand activation (i.e. CXCL-8, CCL-2) among others [2]. Under these influences, macrophages can exhibit an inflammatory phenotype that involves the release of pro-inflammatory mediators, nitric oxide synthase (NOS) or reactive oxygen species (ROS) release, and phagocytosis. As inflammation subsides and wound repair is initiated, macrophages will display a spectrum of anti-inflammatory or resolution-like properties to promote healing and restore homeostasis. The molecular mechanisms that control these responses are not fully elucidated; however, common consensus suggests that myeloid cells possess diverse and flexible functional responses [3], which is influenced in part by epigenetic mechanisms like DNA methylation [4].

DNA methylation is regulated by the combined actions of DNA methyltransferase enzymes (DNMTs), that generate 5-methylcytosine (5mC), a mark typically associated with transcriptional silencing [4]. Conversely, Ten-Eleven Translocation (TET) proteins (TET1, TET2, TET3) regulate DNA de-methylation by oxidizing 5mC to 5-hydroxymethylcytosine (5hmC), which can then trigger passive or active DNA de-methylation. These processes have been linked to myeloid cell differentiation, particularly in progenitor cells and monocytes [5–7] but their role in wound healing in differentiated macrophages remains unclear. Aberrant DNA methylation is associated with chronic inflammation and poor wound healing in conditions such as Crohn’s Disease, a type of inflammatory bowel disease (IBD) that affects the gastrointestinal tract [8–10]. In this context, myeloid cells may modulate both pro- and anti-inflammatory functions during disease progression [11].

While the tumor-suppressive functions of TET enzymes are well established in myeloid and lymphoid malignancies [12–15], their contribution to non-malignant inflammatory diseases remains poorly defined.* In vitro* studies suggest that TET proteins can modulate macrophage cytokine production [16–18], and patient datasets indicate that distinct methylation profiles characterize peripheral monocytes and intestinal tissues in IBD [19] or Crohn’s Disease tissue samples [8, 19, 20]. Moreover, murine models demonstrate that *Tet2* or *Tet3* loss exacerbates colitis or disrupts epithelial homeostasis [21–23]. However, the specific role of TET1, the founding member of the TET family, in regulating innate immune signaling and macrophage function during Crohn’s Disease-associated inflammation remains unknown. Therefore, this study aimed to define how TET enzymes influence macrophage inflammatory programs to assess their potential contribution to the pathogenesis of Crohn’s Disease.

## Materials and methods

### Analysis of publicly available datasets for TET transcriptomic levels and gene enrichment

The TET-specific human gene set METHYLCYTOSINE DIOXYGENASE TET (UNIPROT_A0A023HHK9) [24] was downloaded from the GSEA website (https://www.gsea-msigdb.org) to compare against GSE datasets GSE3365, GSE36807, GSE10616, and GSE117993 available via Gene Expression Omnibus (GEO) [25–28]. Analysis was completed using the computational software available via the Broad Institute.

### Generation of THP-1* TET1* and* TET2* KO CRISPR Cell lines and differentiation into macrophages

The human monocytic leukemia cell line THP-1 (ATCC TIB-202) was used to create CRISPR KO cell lines with guide RNAs specific for *TET1* (AACCUGCAAUGGGUUUACAA) or *TET2* (UUUUCAACACAUAACUGCAG) and transfected to generate homozygous clones with the respective parental negative control (Synthego, California, USA). THP-1 WT, *TET1* KO, and *TET2* KO monocytes cells were cultured and maintained in RPMI-1640 medium supplemented with 10% heat-inactivated fetal bovine serum (Gibco). Cells were differentiated into macrophages using 10 cm^2^ tissue culture dishes in the presence of 5 ng/mL phorbol myristate acetate (PMA, Sigma) overnight, then rested for 3 days following the methods previously published [29]. Differentiated macrophages were scraped and seeded as needed for downstream applications.

### Cytometry staining of surface markers

Unstimulated THP-1 *TET1* KO or *TET2* KO monocytes or macrophages were labeled with CellTrace Violet or CellTrace Far Red proliferation dyes respectively (ThermoFisher), then combined at a 1:1:1 ratio with unlabeled WT monocytes or macrophages as a barcoding approach. This mixed cell ratio was distributed among LEGENDScreen™ plates (BioLegend), stained, washed and fixed according to manufacturer’s protocol. Samples were acquired on a BD FACS Canto II and analysis was completed using FlowJo v10.10 (BD Biosciences). Quantified MFI values from KO cells were normalized to respective WT control and plotted using Spotfire (TIBCO) or GraphPad Prism (Insight Partners) software. Upregulated or downregulated proteins were validated in separate experiments and analyzed via FlowJo using the following antibodies from Biolegend: anti-human CD11b (cat. 393107), anti-human CD14 (cat. 325610), anti-human CD73 (cat. 344003), anti-human CD169 (cat. 346003), anti-human CD235 (cat. 306609), anti-human siglec-9 (cat. 351511), anti-human TLR2 (cat. 309714), and anti-human TLR5 (cat. 394505).

### Phenotypic characterization of WT vs. KO cell lines

Assessment of THP-1 WT or KO monocytes for viability and macrophage differentiation yields were completed with Trypan blue exclusion. For proliferation, THP-1 WT or KO monocytes were labeled with CellTrace Far Red and seeded at 250,000 cells in 12-well plates, then rested for 5 days to assess proliferation rates by cytometry. Phagocytosis and efferocytosis assays used differentiated macrophages seeded in 96-well TC plates at 10,000 cells per well and incubated with pHrodo-labeled E. coli particles (Sartorious) or apoptotic pHrodo labeled Raji cells. Cultures were imaged within 30 min of plating using phase contrast and red or green fluorescence with a 10x or 20x objective, and at 1–2 h intervals with the Incucyte SX5 instrument (Sartorious) for a total of 24 h. Quantification of efferocytosis or phagocytosis was established by acquiring fluorescence over time. Raw values were exported into text format and analyzed with Excel (Microsoft Corporation) and GraphPad Prism. Area under the curve (AUC) values were calculated based on graphing RCU (y-axis) vs. time (x-axis).

### Measurement of inflammatory mediators in LPS-treated macrophages

Macrophages were seeded in 12-well (250,000 cells per well) or 96-well (25,000 cells per well) flat bottom tissue culture plates and stimulated with titrating amounts of E. coli LPS (Sigma) or at 200 ng/mL for overnight stimulation at 37 °C, 5% CO_2_. To assess intracellular signaling pathways the following inhibitors from Tocris Bioscience were used and added at reported cellular IC_50_ concentrations: p38 inhibitor SB 203,580, JNK1/2/3 inhibitor SP 600,125, MEK1/2 inhibitor Trametinib, IKK inhibitor Bay 11-7082, AKT inhibitor MK2206, and PI3K inhibitor Wortmannin. All inhibitors were added at the same time as LPS. Cell-free supernatants were collected and analyzed using the human pro-inflammatory or human chemokine tissue culture kits (Meso Scale Discovery). For CCL-8 analysis, the DuoSet kit was used following manufacturer’s instructions (R&D Systems). For conditioned media experiments, supernatant was collected and frozen in separate batches for analyte analysis or neutrophil migration assessments.

### Neutrophil migration assays

Whole blood from healthy volunteers was requested in K2 EDTA tubes via an internal donor program following established IRB protocols. Total neutrophils were immediately isolated using the direct human neutrophil isolation kit (StemCell Technologies), washed once in PBS/1 mM EDTA and centrifuged gently (250 g’s x 5 min at RT). Final cell pellets were gently resuspended in warmed Macrophage Serum Free media (Gibco) at 0.5 × 10^6^ cells/mL from which 75 µL were dispensed to the apical chamber of a HTS Transwell^®^ 96-well permeable plate (Corning). Prior to dispensing neutrophils, 100 µL of conditioned media from treated and untreated macrophages was added to the basal bottom chamber. Cells were immediately imaged using phase contrast with a 10x or 20x objective, at 15-minute intervals with the Incucyte SX5 instrument (Sartorious) for a total of 24 h. Efficiency of migration was determined by quantifying the total number of cells present in the bottom chamber using a built-in algorithm for cell identification (Sartorious).

### Measurement of intracellular phospho-proteins

Macrophages were differentiated as described above and left untreated or treated with LPS for 10, 15, 30–60 min at 37 °C prior to fixing immediately with 4% PFA (Sigma). Cells were centrifuged (500 g for 5 min at RT), then resuspended in warm BD Phosflow™ Fix Buffer I (BD Biosciences) and incubated at 37 °C for 10 min. Cells were centrifuged again, resuspended in cold BD Phosflow™ Fix Buffer III (BD Biosciences), and incubated at 4 °C for 30 min. Cells were then washed in Perm/Wash buffer (BD Biosciences) and incubated with the following primary antibodies from Cell Signaling at manufacturer’s recommended concentrations: phospho-IRF3 (Ser386, clone E7J8G), Total IRF3 (clone D6I4C), phospho-NFkB p65 (Ser536, clone 93H1), Total NFkB p65 (clone D14E12), phospho-p44/42 MAPK (Erk1/2, Thr202/Tyr204), Total p44/42 MAPK (Erk1/2, clone 137F5), phospho-Akt (Ser473, clone D9E), and Total Akt (clone C67E7). Cells were incubated with primary antibody for 1 h shaking at 4 °C, washed in Perm/Wash buffer, then resuspended with Goat anti-Rabbit IgG Secondary Antibody, Alexa Fluor™ 647 (Life Technologies) shaking for 30 min at 4 °C. After final washes, cells were acquired in the BD FACS Canto II and analysis was completed using FlowJo v10.10 (BD Biosciences).

### RNA-sequencing analysis

Total RNA was isolated using the micro-RNeasy plus kit (Qiagen), assessed for quality on the Nanodrop 8000 (Thermo Scientific), and the RNA integrity assessed via TapeStation 4200 (Agilent). Illumina libraries were prepared with PolyA selection and sequencing at 50 M PE reads. Demultiplexing was performed using bcl2fastq v2.20.0.422 from Illumina. Filtered reads were mapped against the Homo sapiens (human) genome hg38/ using the STAR (v2.5.2b) aligner. Gene expression levels were quantified, using RSEM (v1.3.0) and featureCounts (v1.5.1). Quantification of differential expression was performed on the mapped counts derived from featureCounts. Genes with expression levels that are throughout in noise level (below 10 counts in all samples) were filtered from the data. Differential expression analysis was performed using limma/voom R package. The number of identified genes and transcripts per group was calculated based on p-values < 0.05 and abs(log2FC) > 0.58. Principal component analysis (PCA), correlation analysis, hierarchical clustering, gene ontology (GO), pathway analysis, scatter plots, and volcano plots were performed for the differentially expressed genes in R and Python environment for statistical computing and graphics.

### DNA methylation ELISA

Commercially available ELISAs (Epigentek) were used to quantify the levels of methylcytosine (5mC and 5hmC) in whole genomic DNA samples following the manufacturer’s protocol. Each sample was assayed in technical duplicate from 3 to 4 experimental replicate assays of untreated or LPS-treated (200ng/ml, overnight) THP-1 macrophages.

### 5hmC-sequencing analysis

Reduced representation hydroxymethylation libraries of genomic DNA isolated from LPS-treated macrophages were prepared with the NEBNext Enzymatic 5hmC-seq kit (EM-seq) according to the manufacturer’s recommended protocol with the following modification: instead of random shearing via sonication, genomic DNA was digested with restriction enzyme MspI [30]. All samples were sequenced on the Illumina NovaSeq X Plus platform. FASTQ files were trimmed with cutadapt (v5.0) to remove adaptor sequence with 2 additional bases. The trimmed reads were then aligned to the reference genome GRCh38 with Bismark (v0.24.2) using Bowtie 2 (v2.5.4) for alignment. Genomic arithmetic operations were performed using Bedtools (v2.31.1). CpG sites were destranded with bsseq package (v.1.36.0) in R (v4.3.3). We required a coverage threshold greater or equal to 10 and less than 400. We used the methylSig package (v. 1.16.0) to call hmC differences using a threshold of greater than 10% differential hydroxymethylation. Cell lines were analyzed for unbiased sample clustering by principal component analysis and differential 5hmC detection. Genome-wide and CpG-island annotations were drawn from HOMER and the UCSC Genome Browser, respectively. Transcription factor motifs from JASPAR (v 2024) were searched with fimo of MEME Suite (v. 5.5.8) on 50nt-binned genome. Bins that contained at least one CpG that was sufficiently covered comprised the background set of CpGs. To look for enrichment of motifs on bins that contained at least one hyper- or hypo-hydroxymethylated CpG we employed hypergeometric tests, correcting p-values to FDR values and requiring a threshold of FDR < 1% to call a motif as significantly enriched. 5hmC loci were annotated to genes, and gene set enrichment of differential 5hmC-annotated genes was performed again using hypergeometric testing against the KEGG, Hallmark and Reactome gene sets from the Broad Human Molecular Signatures Database (MSigDB).

### Analysis of Crohn’s Disease patient samples

Frozen peripheral mononuclear cells (PBMCs) with confirmed diagnosis of Crohn’s Disease or designated as healthy were sourced from Sanguine Biosciences or BioIVT and stored in liquid nitrogen tanks until ready for use. Cells were recovered by rapid thaw and assessed for viability and yield prior to any studies. For RNA analysis, 1–2 million cells were lysed in RLT+ (Qiagen) then processed for TaqMan analysis as described in the RNAseq section. The mRNA expression levels of the target genes, *TET1 (hs00286756_m1)*,* TET2 (hs00286756_m1)*,* TET3 (hs00379125_m1)*,* PTEN (Hs02621230_s1)*,* RMB38* (Hs00955734_g1) and *CCL8* (hs04187715_m1) were quantified on cDNA-based RNA using Applied Biosystems TaqMan Gene Expression Assays. Analysis of Ct counts was performed using the fold-change expression based on the comparative delta-Ct method and normalized to an internal housekeeping gene.

To assess levels of CCL-8 release, cells were resuspended in cell media (RPMI, 10% FBS) and dispensed at 100,000 cells per well in a 96-well TC plate. LPS was added at 200 ng/mL and incubated overnight. Supernatant was assessed for TNF-α, IL-6, CCL-2, or CCL-8 levels using MSD or DuoSet ELISA kits.

### Analysis of bulk or scRNA-seq transcriptome in Crohn’s Disease tissue

Raw sequencing reads from published bulk or scRNA-seq were downloaded and processed as described above. Samples for bulk sequencing included *N* = 43 healthy controls and *N* = 63 Crohn’s Disease ileal biopsies from biologically naïve pediatric patients [31]. Samples for scRNA-seq included biopsies from *N* = 16 biologic-naïve patients and *N* = 3 healthy controls allowing analysis of transcriptomic expression at the single-cell resolution [32].

### Statistical analyses

Statistical analyses and graphing were performed using GraphPad prism 10 and/or Excel (Microsoft Corporation). For two groups, statistical significance was determined using the student’s t-test. For three or more groups, statistical significance was determined using one-way or two-way analysis of variance (ANOVA). All results are expressed as mean ± SEM, with individual data points illustrated in bar graphs. Statistical significance was defined as ns: no significance, *p < 0.05, ** *p* < 0.01, *** *p* < 0.001, and **** *p* < 0.0001 for all statistical tests.

### Figure schematics

Schematics were created using BioRender.com.

## Results

### Aberrant DNA methylation in IBD patient samples is correlated with downregulated* TET1* expression

**Fig. 1:**
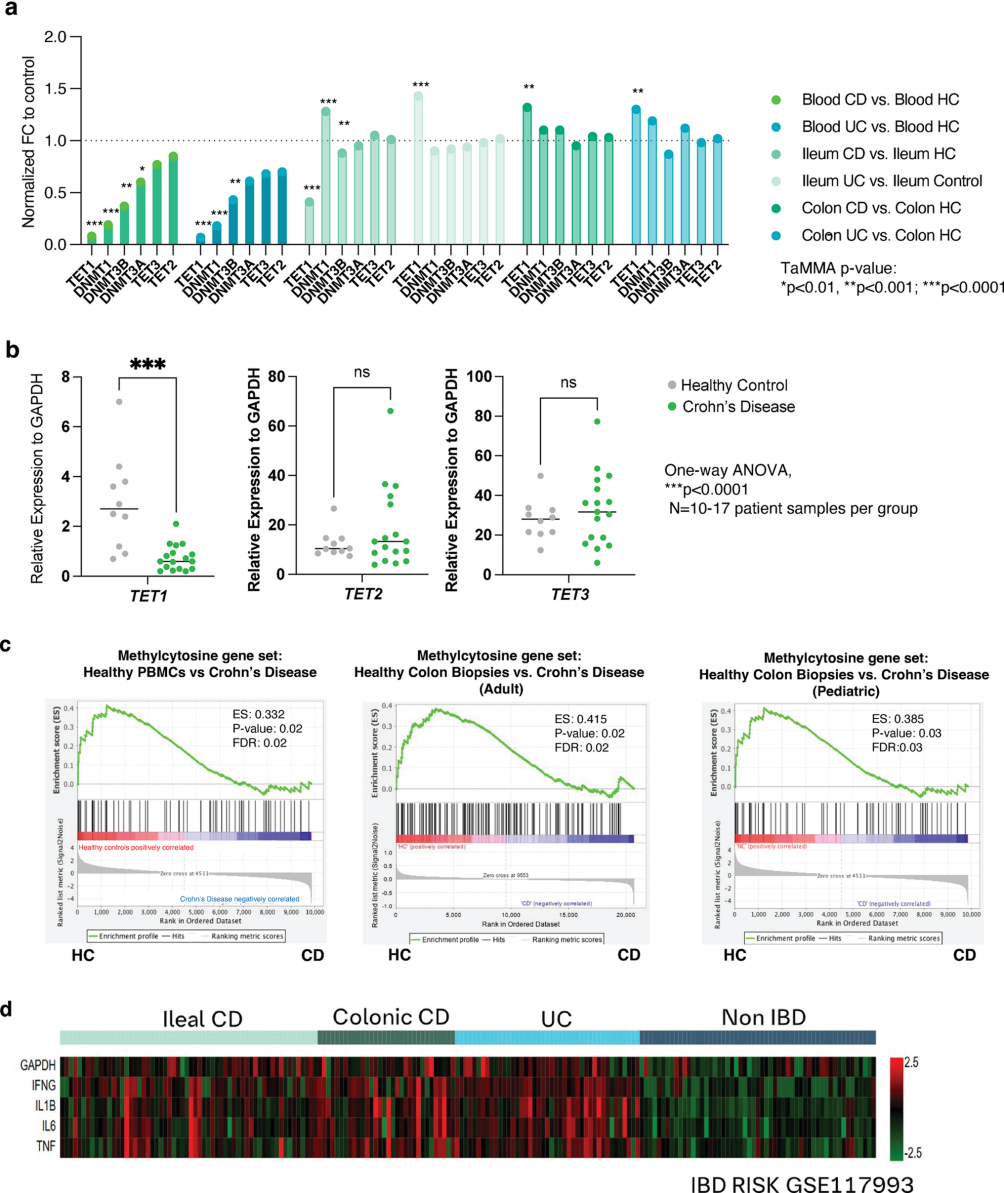
Decreased* TET1* expression correlates with upregulation of inflammatory mediators in IBD patient samples. (**a**) Differential gene expression analysis from the IBD Transcriptome online platform. (**b**) Gene set enrichment analysis of methylcytosine dioxygenase genes expressed in healthy control samples versus Crohn’s Disease (CD) samples of PBMCs (left panel), adult colon biopsies (middle panel) and pediatric colon biopsies (right panel). (**c**) Inflammatory mediators are transcriptionally upregulated in tissue sections in IBD patient samples

Published studies have identified aberrant DNA methylation patterns in peripheral monocytes [19] or intestinal mucosa lesions [8] derived from patients with Crohn’s Disease. More specifically, roles for DNA de-methylases such as *TET1* and *TET2* have been described in IBD leukocytes [33, 34] however, *TET3* appears to play a role in intestinal epithelial cells [22]. To further investigate links between TET1, TET2, or TET3 and Crohn’s Disease, we conducted an assessment in the IBD Transcriptome and Metatranscriptome Meta-Analysis online platform (IBD TaMMA: https://ibd-meta-analysis.herokuapp.com) providing quick visualization and analysis for gene expression in IBD tissues [35]. Differential gene expression analysis identified *TET1* as the most statistically significant modulated gene in comparison to *TET2* or *TET3* in Crohn’s Disease samples of blood, ileum and colon tissue (Fig. 1a). We corroborated these findings in peripheral mononuclear cells from healthy controls and Crohn’s Disease patients (acquired from Sanguine Biosciences). Our cohort showed that *TET1* is significantly decreased in the patient sample group with Crohn’s Disease without any significant change in *TET2* or *TET3* expression (Fig. 1b). In addition, publicly available RNAseq data from isolated human peripheral blood mononuclear cells (PBMCs) [25] was investigated via gene set enrichment analysis (GSEA) for expression of a methylcytosine TET transcriptomic signature [24] (Molecular Signatures Database). Results demonstrated a decreased gene set enrichment in patients with Crohn’s Disease (Fig. 1c, left). Similarly, other publicly available datasets [26–28] from diseased tissue resections were assessed for TET gene set enrichment (Fig. 1c, middle and right) and expression of characterized inflammatory mediators pertinent to IBD (Fig. 1d). These results verified published literature confirming elevated levels of inflammatory mediators is associated with a decrease in TET activity in Crohn’s Disease samples.

### Characterization of* TET1* or* TET2 *deficient* THP-1* myeloid cells reveals unique roles in macrophage differentiation

The finding that reduced *TET1* expression was more strongly associated with Crohn’s Disease prompted us to conduct mechanistic* in vitro* studies using *TET1* KO myeloid cells to determine their role in macrophage effector function. *TET2* KO cells were also generated as a control due to the extensive literature surrounding a role for TET2 in pro-inflammatory myeloid cell signaling [18, 21, 36, 37].

**Fig. 2:**
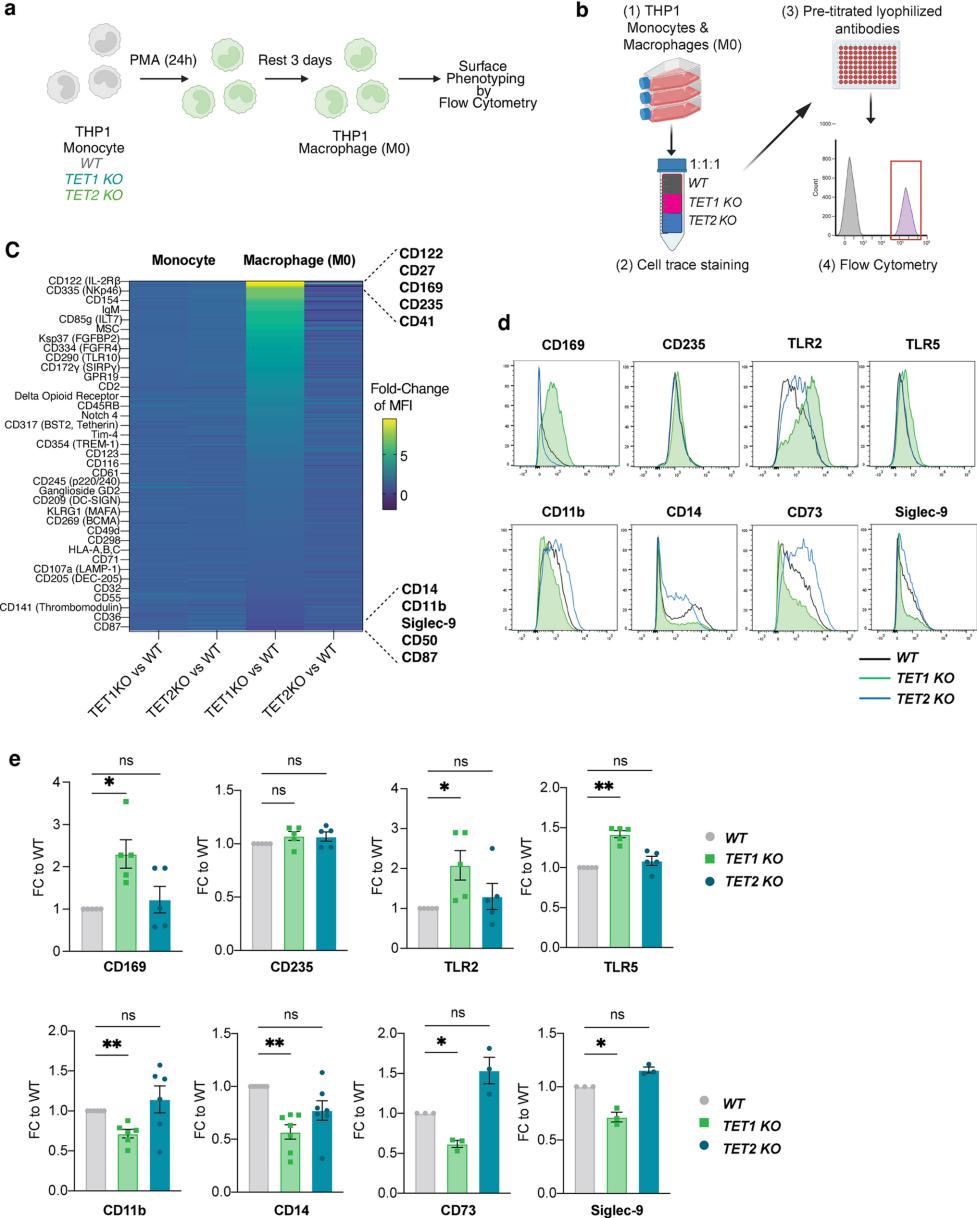
*TET1* deficiency in macrophages alters their functional surface marker phenotype. (**a**) Schematic representation of CRISPR KO THP1 myeloid cells monocytes differentiated into macrophages (M0) upon PMA treatment. (**b**) Schematic representation of simultaneous surface staining using LEGENDScreen^™^ (BioLegend) for monocytes or M0 macrophages. (**c**) Heatmap summary of fold change in mean fluorescence intensity (MFI) of surface markers between KO versus WT control THP1 monocytes or M0 macrophages. (**d**) Representative histograms of M0 macrophages and (**e**) summary of fold-change in downregulated or upregulated receptors identified as hits in the screen and additional markers relevant to IBD (TLR2, TLR5)

The THP-1 monocytic cell line, a well-established model for investigating monocyte and macrophage responses, was used to create CRISPR-mediated knockout of *TET1* and *TET2* and to examine the resulting functional outcome [29]. We first confirmed decreased expression and loss of 5hmC in KO macrophage (M0) cell lines (Fig. S1a-b). Next, we performed an analysis of surface markers in untreated monocytes vs. macrophages (M0) where we applied a high-throughput cell surface staining approach using a LEGENDScreen^™^ assay kit (BioLegend) (Fig. 2a). This enabled simultaneous staining of 364 surface proteins that were previously titrated for their optimal concentrations, including corresponding isotype controls. THP-1 monocytes and differentiated macrophages were analyzed in an unstimulated state to understand basal levels of expression. To minimize inconsistencies in staining between samples, *TET1* and *TET2* KO cells were stained with violet or red CellTrace dyes, respectively, and mixed in equal concentrations with unlabeled WT cells as a cell line identification method prior to staining with the PE-labeled panel of antibodies (Fig. 2b and Fig. S1c-e). The mean fluorescence intensity (MFI) of staining for each surface protein was normalized to its corresponding WT signal allowing comparison of *TET1/WT* to *TET2/WT* ratios. Minimal differences were noted in *TET1/WT* vs. *TET2/WT* monocytes, however significant changes were observed in *TET1/WT* macrophages (Fig. 2c). We validated the most upregulated and downregulated surface markers in separate cytometry experiments (representative histograms shown in Fig. 2d) and confirmed significant upregulation of CD169, a marker of tissue resident macrophages [38] and downregulation of CD14, a myeloid maturation marker that acts as a receptor for lipopolysaccharide (LPS) [39] in *TET1* KO THP-1 macrophages compared to WT controls (Fig. 2e). In addition, we measured receptors known to impact Crohn’s Disease pathogenesis that were not identified by our initial screen including TLR2 and TLR5, which showed increased expression in the *TET1* deficient macrophages (Fig. 2e). Additional analysis of viability, macrophage differentiation yield, and proliferation rate showed no differences between *WT* vs. *TET1* or *TET2 KO* cells (Fig. S1f-h) suggesting that the surface marker expression changes reflected differential effector function potential and not a growth defect.

### Inflammatory responses are augmented in* TET1* and* TET2 *deficient THP-1 macrophages* in vitro*

**Fig. 3:**
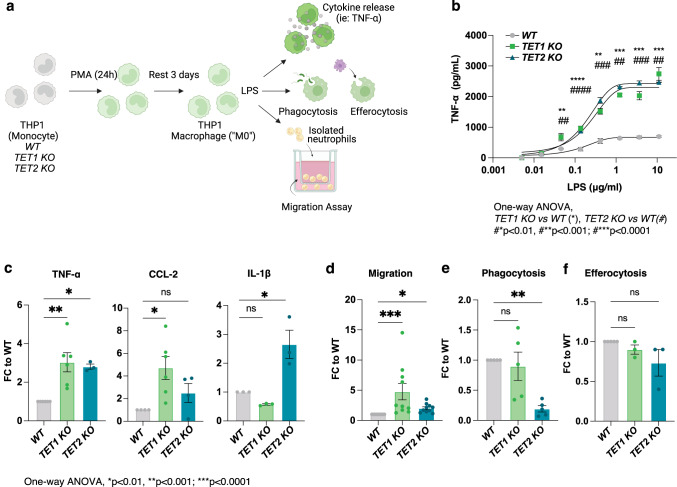
*TET1* or* TET2* deficiency in macrophages causes overlapping and unique increases in the secretion of inflammatory mediators and alters effector function* in vitro*. (**a**) Schematic representation of macrophage functional assays to assess cytokine or chemokine secretion, leukocyte migration, phagocytosis, and efferocytosis. (**b**) TNF-α secretion in response to stimulation via LPS titration from *TET1* or *TET2* KO THP1 macrophages. (**c**) Summary of secreted inflammatory mediators measured in response to LPS (200ng/ml). (**d**) Migration (**e**) phagocytosis and (**f**) efferocytosis capacity of WT versus *TET1* or *TET2* KO cells. Data representative or summarized from N *≥* 3 separate experiments; One-way ANOVA, *p < 0.01, **p < 0.001, ***p < 0.0001

Gut dysbiosis is a common characteristic found in patients with IBD whereby myeloid cells are known to play a central role in innate inflammatory responses [40]. To assess the role of TET1 and TET2 in this context, we selected LPS as an inflammatory stimulus due to its disease-relevance in Crohn’s Disease [41]. THP1 cells differentiated into macrophages were exposed to LPS in a dose-dependent titration and incubated overnight for analysis of pro-inflammatory chemokine and cytokine production. Innate functional responses (phagocytosis or efferocytosis), and impact on chemotaxis (migration of leukocytes) were also measured (Fig. 3a). Both *TET1* and *TET2* deficient THP1 macrophages generated increased levels of TNF-α in a dose-dependent manner in the supernatant after overnight incubation with LPS (Fig. 3b). The most significant difference was observed at 0.2 mg/mL of LPS treatment and was chosen as a standard LPS concentration for all further assays. An expanded cytokine and chemokine panel was later included to assess additional inflammatory mediators in response to LPS and identified CCL-2 (MCP1) and IL-1β as analytes with exaggerated production from *TET1* or *TET2* deficient macrophages, respectively (Fig. 3c). The augmented responses of IL-1β observed upon LPS stimulation of *TET2*-deficient macrophages was consistent with previous studies [17, 42]. These data are the first to associate *TET1* as a negative regulator of CCL-2 production by macrophages.

CCL-2 is a powerful chemoattractant known to be elevated in patients with IBD [43–45], is linked to the development of fibrosis [46–48], and contributes to the pro-inflammatory processes underlying IBD pathogenesis [49]. We therefore sought to assess the influence of conditioned media from overnight treated and untreated *TET1* and *TET2* deficient THP-1 macrophages on leukocyte migration. We specifically assessed neutrophil migration since recent analyses have identified these populations as critical contributors of inflammation via the release of neutrophil extracellular traps (NETs) [50] or by increased interactions with activated fibroblasts isolated from IBD tissue resections [51]. Our results showed a significantly increased migration capacity of neutrophils to the conditioned media from both untreated *TET1* and *TET2* KO overnight cultures, however the fold-change was greater for *TET1* KO (Fig. 3d).

 Efferocytosis and phagocytosis are key effector functions of macrophages. Interestingly, while phagocytosis was impaired by loss of *TET2* (Fig. 3e) as previously demonstrated in neutrophils with *TET2*-deficiency [52, 53], efferocytosis was not affected, nor was there any difference noted in these assays between *TET1* deficient macrophages and *WT* control cells (Fig. 3e-f). These studies suggest *TET1* plays a more important role in migration than *TET2*, while the latter regulates phagocytosis activity of macrophages.

### *TET1* and* TET2* deficient macrophages exhibit an elevated type I interferon transcriptional signature

**Fig. 4:**
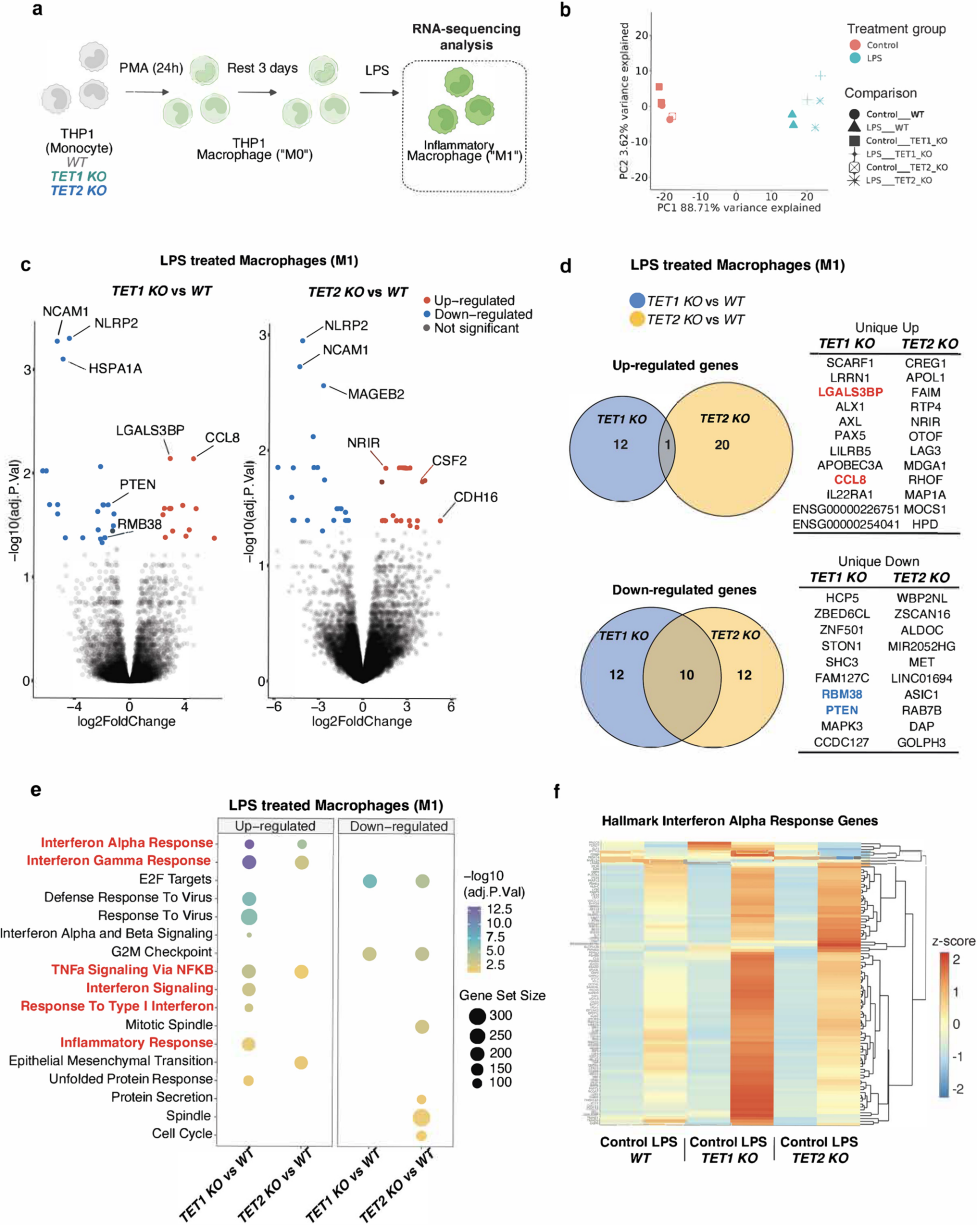
Both *TET1* and *TET2* deficient inflammatory macrophages exhibit an elevated type I IFN transcriptional signature. (**a**) Schematic representation of THP1 macrophage cells used for RNAseq analysis. (**a**) Principal component analysis (PCA) and (**c**) Volcano plots of LPS treated *TET1* KO vs. WT (left) or LPS treated *TET2* KO vs. WT (right) cells showing upregulated (red) and downregulated (blue) transcripts in KO vs. WT cells. Differential gene expression was assessed using DESeq2, with significance defined as adjusted *p* *≤* 0.05 and log₂ fold-change *≥* 0.5. (**d**)) Venn diagrams depicting number of significant differentially expressed genes unique to *TET1* or *TET2* KO. (**e**) Gene ontology (GO) enrichment analysis reveals significant upregulation of inflammatory and type I interferon pathways in KO cells. (**f**) Heatmaps representation of Hallmark IFN alpha response genes displayed as raw tpm expression of differentially expressed genes across treated and untreated macrophage genotypes

Given that *TET1* KO and *TET2* KO macrophages exhibited amplified but unique responses to inflammatory stimuli, a bulk RNA sequencing study was conducted to elucidate the pathways and genes implicated in their response. THP-1 macrophages were stimulated overnight as described above and processed for RNA isolation (Fig. 4a). Principal component analysis showed variance in untreated vs. LPS-treated samples confirming the greatest differences were associated with stimulation (Fig. 4b), however, to identify differentially expressed genes specific to *TET1* or *TET2* deficient macrophages, we compared these samples against LPS-treated WT controls and identified unique dysregulated genes in either *TET1* or *TET2* KO macrophages with thresholds set at p *≤* 0.05 and log2FC *≥* 0.58 (Fig. 4C-D). Amongst the top upregulated genes in *TET1* KO THP-1 macrophages were *CCL8* and *LGALS3BP*, both of which have been described as playing pro-inflammatory roles in inflammatory diseases [54–57]. Whereas genes encoding RBM38 and PTEN, known to negatively regulate AKT and ERK, proliferation, and inflammation [58–60], were significantly downregulated in *TET1* KO macrophages (Fig. 4d). Analysis of human gene sets (Reactome, Hallmark, and GO) identified several pathways differentially expressed in either or both *TET1* and *TET2* deficient macrophages (Fig. 4e-f), including Type I Interferon (IFN) response genes, which were more significantly upregulated in *TET1* deficient macrophages (Fig. 4e-f). Published data has elucidated a role for AKT activation in Type I IFN responses [61], so while our results suggest both TET1 and/or TET2 regulate inflammatory gene signatures, *TET1*-deficiency causes a stronger upregulation of type I-IFN genes.

### *TET1* and* TET2* regulate unique CpG hydroxymethylation signatures in macrophages

**Fig. 5:**
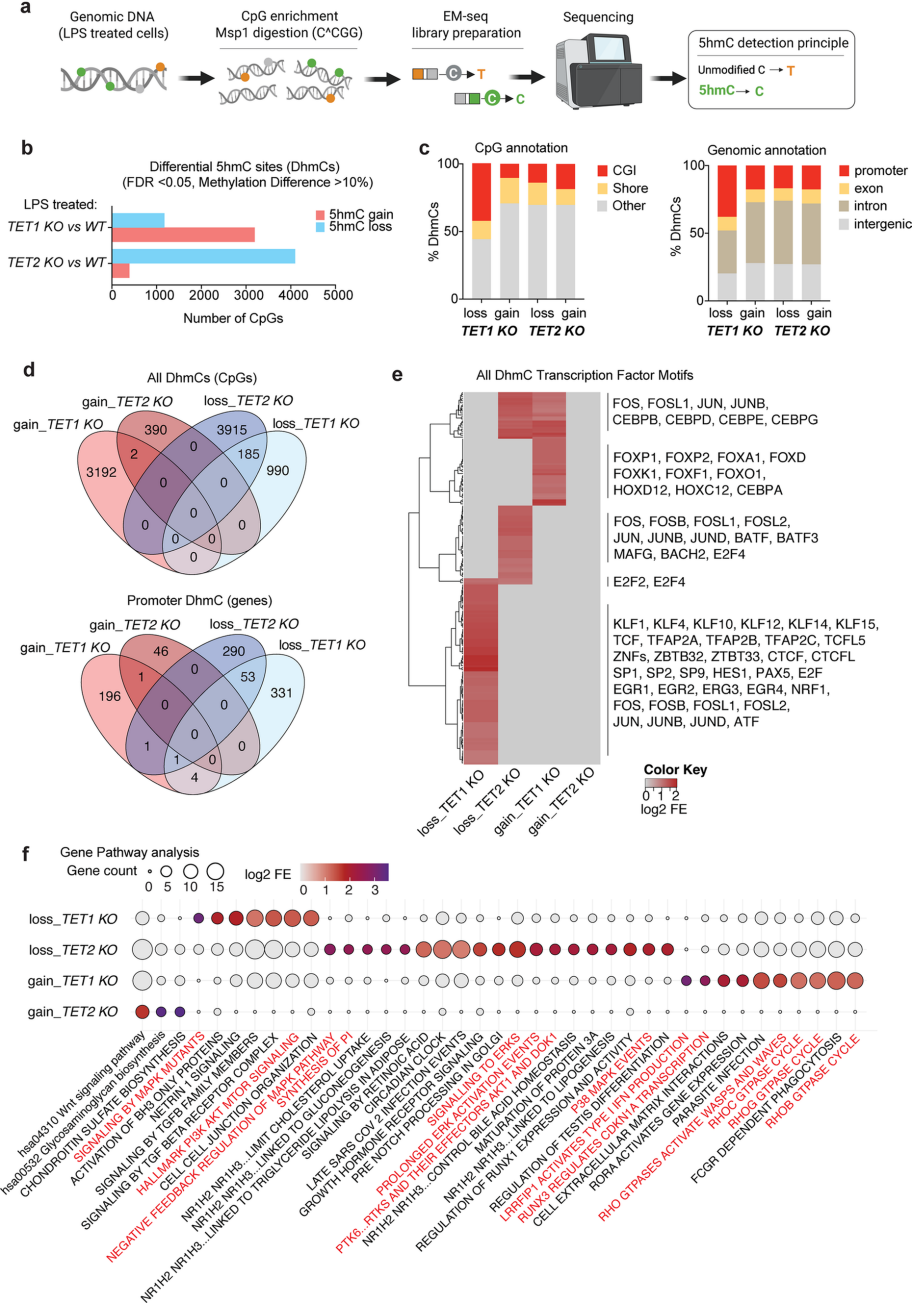
*TET1* and* TET2* regulate unique CpG hydroxymethylation signatures in macrophages. (**a**) Schematic of the 5hmC profiling workflow. Genomic DNA isolated from LPS-treated macrophages was subjected to MspI digestion (cut site C^CGG), EM-seq library preparation, and high-throughput sequencing to detect 5hmC at CpG resolution. (**b**) Quantification of differential hydroxymethylated CpG sites (DhmCs) identified in *TET1* and *TET2* KO macrophages compared to *WT* (FDR < 0.05, methylation difference > 10%). Bar plots show the number of CpGs exhibiting 5hmC loss or gain in each genotype compared to *WT*. (**c**) Genomic distribution of DhmCs (percent distribution of 5hmC gain and loss) across CpG islands (CGI), shores, and other regions (left panel) or annotated to promoter, exon, intron, and intergenic regions (right panel). (**d**) Venn diagram of all DhmCs and genes with promoter-associated DhmCs. (**e**) Transcription factor motif enrichment analysis of DhmCs. Heatmap depicts log2 fold enrichment (FE) of motifs associated with 5hmC gain or loss. (**f**) Pathway enrichment analysis of genes with DhmCs. Significantly enriched pathways are shown, with gene count and log2 fold enrichment indicated

To determine how loss of *TET1* or *TET2* affects 5hmC abundance and distribution following LPS stimulation, we performed CpG-enriched Enzymatic Methyl sequencing (EM-seq) [62] (Fig. 5a). Differential hydroxymethylated CpG sites (DhmCs; FDR < 0.05, methylation difference > 10%) were identified for each knockout relative to WT. While quantitation by ELISA showed lower levels of 5hmC and 5mC in LPS-stimulated KO THP-1 macrophages compared to WT (Fig S2 a), EM-seq of the CpG-enriched genome showed that *TET2* KO specifically caused widespread 5hmC loss (4100 CpGs) and a small subset of gains (392 CpGs), whereas *TET1* KO showed fewer losses (1175 CpGs) compared to gains (3194 CpGs) (Fig. 5b, Fig. S2 b). Genomic annotation of DhmCs revealed that *TET1 KO* 5hmC losses were more strongly enriched within CpG islands (CGIs) and promoters (where most CGIs reside) (Fig. 5c) compared to *TET2 KO.* Importantly, we find the vast majority of CpGs are uniquely regulated by either TET1 or TET2, with only a small fraction of 5hmC losses overlapping at the same CpG sites within the promoters of target genes (Fig. 5d, Fig.S2 c). These finding are consistent with chromatin mapping studies that indicate TET1 and TET2 occupy and regulate different regions of the genome [63]. TET1 is primarily associated with CpG-rich promoter regions and transcription start sites whereas TET2 tends to be more heavily involved in regulating 5hmC levels in gene bodies, exon boundaries of highly expressed genes, and distal regulatory elements (enhancers) [64, 65]. The difference in localization is partly due to the presence of a CXXC domain in TET1, which allows it to directly bind to DNA, whereas TET2 lacks this domain and is likely recruited via other mechanisms such as protein-protein interactions [64, 65].

Transcription factor motif enrichment analysis of all DhmCs demonstrated strong representation of AP-1 members (FOS and JUN), specifically at CpGs with 5hmC loss in either *TET1* or *TET2* KO cells (Fig. 5e). Most C/EBP family motifs were unique to *TET2* loss, whereas *TET1*-dependent changes showed enrichment for FOX, HOX and KLF motifs, which are linked to developmental and lineage specification programs, including macrophage polarization, phagocytosis, migration, and tissue resident identity [66–68]. These patterns suggest that *TET1* and *TET2* influence distinct regulatory networks in LPS-treated macrophages.

Pathway analysis of genes with DhmCs further highlighted divergent functional signatures (Fig. 5f). Both *TET1* and *TET2* KO 5hmC losses were enriched in gene pathways related to MAPK and PI3K/AKT signaling. *TET2* KO 5hmC losses were also associated with genes involved in metabolic regulation, whereas *TET1* KO 5hmC gains were enriched within genes involved in type I IFN production and RHO GTPase signaling, suggesting these pathways exhibit aberrant activation upon loss of *TET1* that correlate with enhanced IFN alpha gene signatures detected by RNAseq. Collectively, these results demonstrate that *TET1* and *TET2* differentially shape the hydroxymethylome of macrophages following LPS stimulation, targeting genes with overlapping and non-redundant roles in inflammatory signaling.

### *TET1* deficiency disrupts expression of* PTEN* and* RBM38* and increases* ERK1/2* signaling

PTEN is a well-established tumor suppressor playing a central role in the regulation of inflammation, cell growth, survival, and genomic stability [69]. RBM38 has been described to stabilize and upregulate PTEN expression [70], directly inhibiting PI3K-AKT activity, which in turn can regulate IκB kinase (IKK) leading to modulation of the NF-κB pathway [71]. The enhanced inflammatory responses observed upon LPS stimulation in *TET* deficient macrophages prompted us to investigate the role of TLR4/MyD88-dependent and independent signaling pathways and their association with chemokine dysregulation in more detail.

**Fig. 6:**
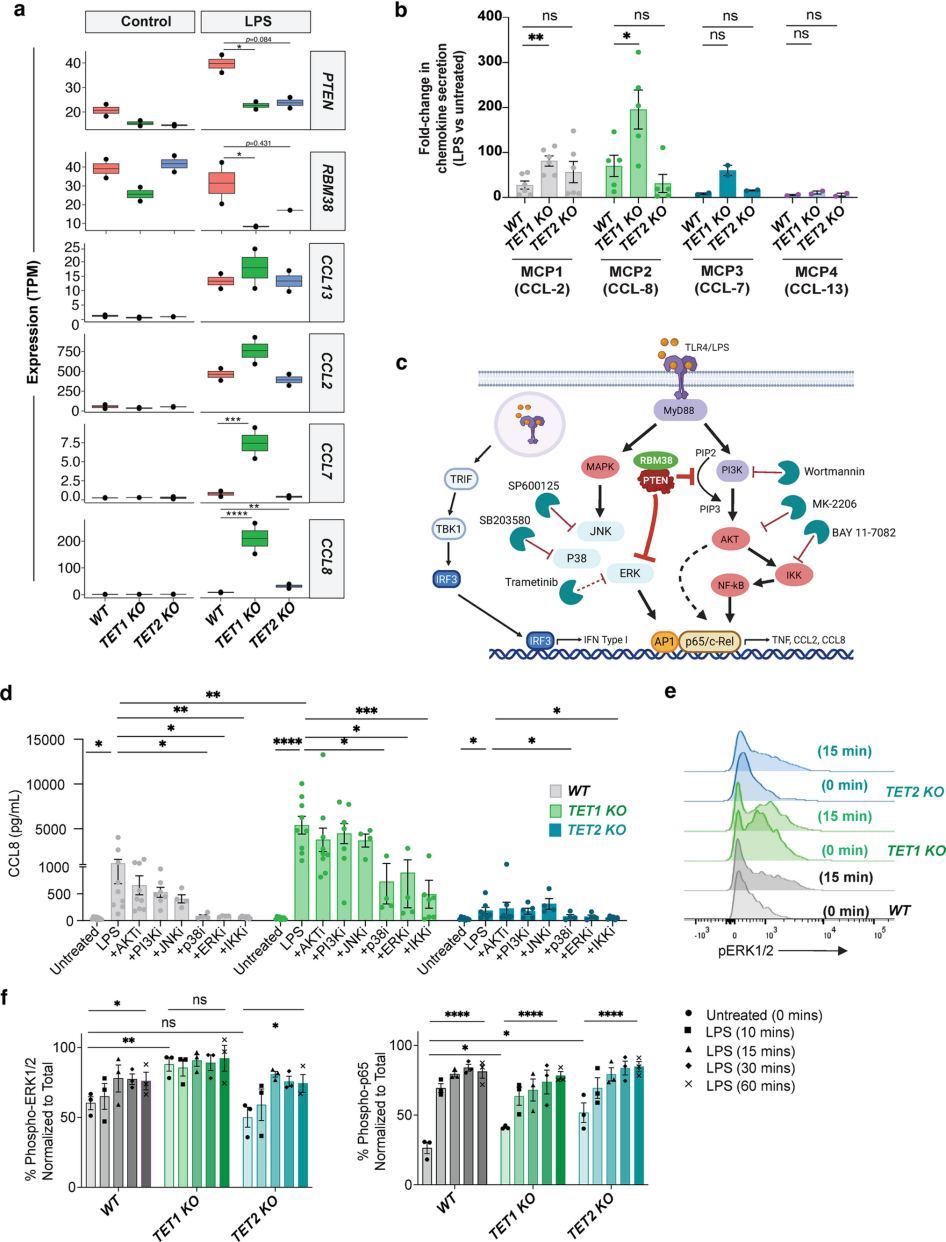
*TET1 *deficiency drives aberrant chemokine upregulation via* MAPK* and NF-kB pathways. (**a**) Transcriptional analysis of *PTEN*, *RBM38* and selected chemokines in LPS-treated *TET1* and *TET2* KO macrophages in comparison to WT control. (**b**) Summary of chemokine levels analyzed in LPS-treated supernatant from macrophage cells normalized to untreated control. (**c**) Schematic representation of AKT, IKK, NF-kB, MAPK, and TRIF signaling pathways and inhibitor screening. (**d**) CCL-8 levels secreted in response to LPS treatment of macrophages co-cultured with PI3K, AKT, JNK, p38, ERK1/2, and IKK/NF-kB inhibitors at their reported IC_50_ concentrations. (**e**) Representative histograms of ERK1/2 phosphorylation at basal levels (untreated) and 15 min post LPS stimulation. (**f**) Percent phosphorylated of ERK1/2 and p65 normalized to total protein in a time-course of LPS treated macrophages. Statistical significance was determined using unpaired t-tests. ns: no significance, *p < 0.05, ** *p* < 0.01, *** *p* < 0.001, and **** *p* < 0.0001 for all statistical tests

Expression analysis showed that despite a similar trend in *TET2* KO THP-1 macrophages, downregulation of both *PTEN* and *RBM38* was only significant with *TET1* deficiency (Fig. 6a). Downregulation of these genes correlated with upregulation in chemokine gene expression (e.g. *CCL13*, *CCL2*, *CCL7* and *CCL8*) and protein production in *TET1* deficient macrophages (Fig. 6a-b). We next performed a series of experiments to assess the role of PI3K-AKT-NF-κB (MyD88-dependent), and TRIF-IRF3 (MyD88-independent) LPS responsive pathways, in addition to MAPK signaling and their role in chemokine production by *TET* KO macrophages (Fig. 6c). First, we examined the effects of PI3K, AKT, IKK/NF-κB, ERK1/2, JNK, and p38 inhibition using Wortmannin, MK2206, BAY11-7082, Trametinib, SB203580, and SP600125 respectively, at standard manufacturer’s recommendedconcentrations. Upon LPS stimulation, no significant difference in CCL-8 production was detected by PI3K, AKT, or JNK inhibition. However, IKK/NF-κB, p38 and ERK1/2 inhibition effectively suppressed CCL-8 release confirming their roles in this pathway (Fig. 6d). Our transcriptomic data did not show changes at the gene expression level for these pathway genes (Fig. S3 a, b) suggesting *TET1*-deficiency in macrophages may result in increased intracellular signaling strength rather than altered transcription. Next, we sought to measure the percent of phosphorylated proteins relevant to these pathways. Assessment of AKT, ERK1/2, IRF3 and NF-κB phosphorylation in a time course of LPS stimulation showed that ERK signaling was strongly and significantly activated already at baseline only in *TET1* KO cells. Phosphorylation of p65 (NF-κB) was significantly elevated at baseline in both *TET1* and *TET2* KO cells, while IRF3 phosphorylation increased to the same degree in all cells, and only at later time points, as previously described [72] (Fig. 6e-f, Fig. S3 c). Given that elevated CCL-2 and CCL-8 production in response to LPS occurred exclusively in *TET1* KO macrophages (consistent with RNA-seq transcriptional changes), these data suggest that heightened basal ERK1/2 phosphorylation in combination with NF-κB may be enhancing the downstream production of these chemokines.

### Patient-derived Crohn’s disease samples exhibit decreased basal levels of* TET1*,* RMB38*, and* PTEN* expression, and increased CCL-8 expression and secretion in response to LPS stimulation

**Fig. 7:**
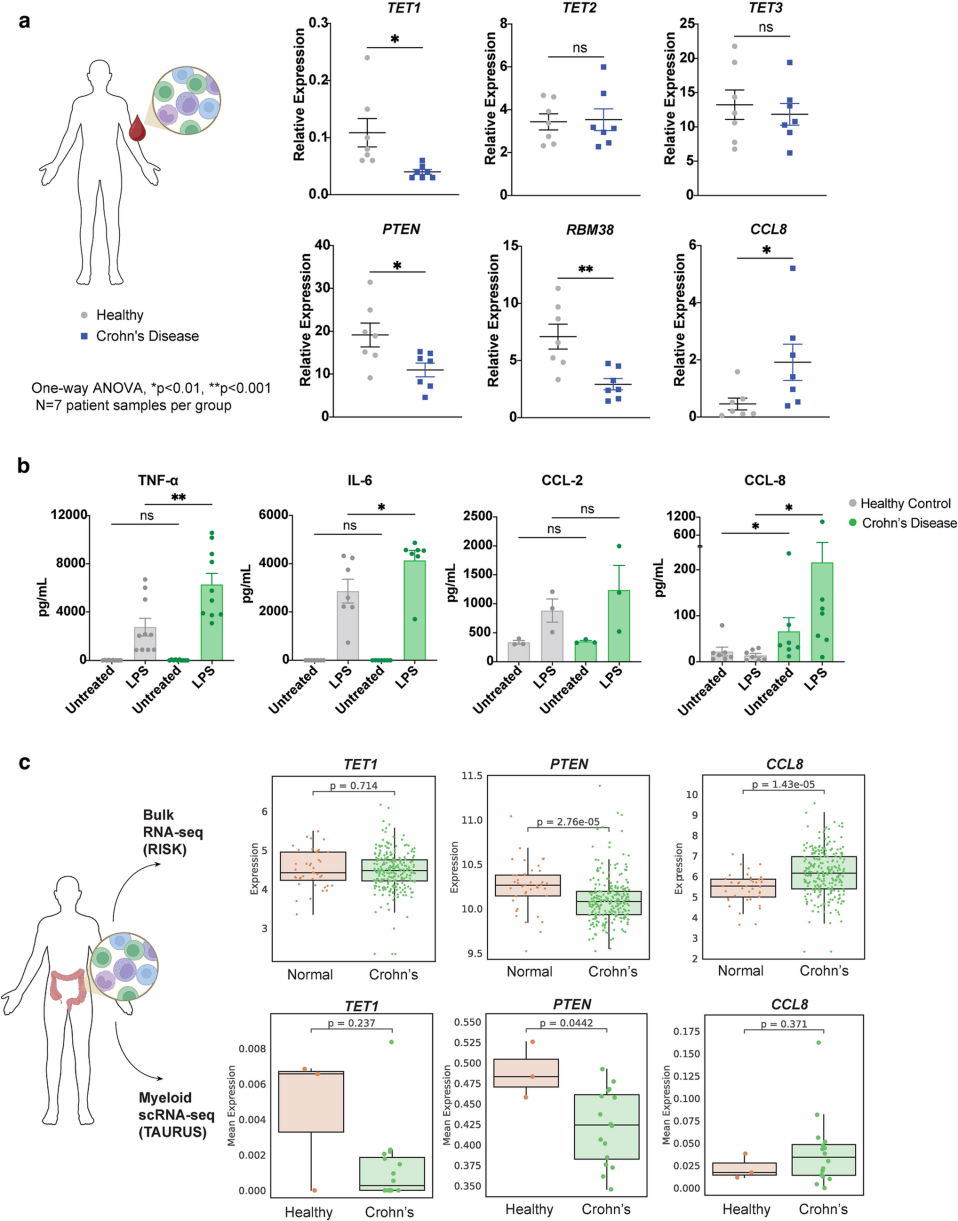
Crohn’s disease patient samples have decreased basal levels of* TET1* expression and exaggerated CCL-8 secretion in response to LPS stimulation. (**a**) Quantitative rtPCR for *TET1*, *TET2*, *TET3*, *RMB38*, *PTEN*, and *CCL8* expression in peripheral blood mononuclear cells (PBMCs) isolated from healthy controls and Crohn’s Disease (CD) patients. (**b**) Levels of TNF-α, IL-6, CCL-2, and CCL-8 secretion from PBMCs of healthy controls versus patients with Crohn’s Disease upon overnight stimulation with 200 ng/mL LPS (*p < 0.01, **p < 0.001). (**c**) Analysis of *TET1*,* PTEN and CCL8* mRNA expression levels in normal vs. Crohn’s Disease total ileal tissue sections (upper panels) and myeloid cells (lower panels)

To further explore links between *TET1* and increased CCL-8 production, we measured matching mRNA expression levels of *TET1*, *TET2*, *TET3*,* RBM38*,* PTEN*, and *CCL8* in peripheral mononuclear cells from a new cohort of healthy controls and patients with Crohn’s Disease. *TET1* was found to be significantly downregulated in the patient sample group with Crohn’s disease without any significant changes in *TET2* or *TET3* expression (Fig. 7a), corroborating the initial observation in a previous set of patient samples (Fig. 1b). Furthermore, *PTEN* and *RBM38* levels were also reduced, while *CCL8* mRNA levels were upregulated in samples from patients with Crohn’s Disease (Fig. 7a). Mononuclear cells from the same samples were stimulated overnight with LPS and supernatants were processed the next day. Our results showed increased secretion of TNF-α, IL-6, CCL-2 and CCL-8 from the Crohn’s Disease samples (Fig. 7b). Notably, levels of CCL-8 were also significantly elevated at baseline.

Crohn’s Disease is characterized by inflammation throughout the intestine; hence, we sought to analyze tissue resections from patients that had undergone corrective surgery due to disease complications. Bulk RNA-seq analysis from a pediatric cohort (RISK) [31] and scRNA-seq transcriptomic analysis of myeloid cells within ileal lesions (TAURUS) [32] was used to assess transcriptomic levels of *TET1*, *PTEN* and *CCL8*. Adjacent normal tissue served as controls in the RISK study, and tissue sections from related surgeries (healthy controls but not diagnosed with Crohn’s Disease) were used for the TAURUS study groups. In both analyses, significant downregulation of *PTEN* was observed in total tissue or myeloid cell samples from patients with Crohn’s Disease (Fig. 7c). Though not statistically significant in these data, *TET1* showed a trend toward downregulation in the tissue resident myeloid cells, while *CCL8* was found to be significantly increased in the bulk-RNA seq analysis, and trending towards upregulation in the myeloid compartment. Based on our knockout studies and supporting evidence from multiple patients cohorts of Crohn’s Disease, our findings collectively suggest that loss of TET1 activity may be causative of downregulated levels of *PTEN* in the intestinal mucosa, promoting a pro-inflammatory state driven by increased production of chemokines like CCL-2 and CCL-8, as well as type I IFN-related genes.

## Discussion

Our findings have identified* TET1* as an important regulator of macrophage effector function and chemokine signaling. We show that loss of *TET1* expression is a hallmark of IBD and specifically associated with dysregulated DNA methylation in PBMCs [19] and intestinal tissue samples from patients with Crohn’s Disease [8, 20]. These findings distinguish TET1 as a functionally distinct regulator from the more frequently implicated TET2 in myeloid cell biology. *TET2* deficiency is known to block mature myeloid cell differentiation leading to an accumulation of immature myeloid cells, and monocytosis [73, 74]. *TET2* loss in tumor-associated macrophages also shifts their function from immunosuppressive to pro-inflammatory [75]. We now show that TET1 also exerts a unique role in differentiated macrophages. *TET1* deficiency led to reduced expression of both *PTEN* and *RBM38* (its upstream stabilizing partner). Exacerbated proinflammatory type I IFN and chemokine expression levels in response to LPS stimulation also correlated with increased basal ERK phosphorylation and higher surface expression of TLR2 and TLR5 only in *TET1* deficient macrophages, suggesting increased susceptibility to innate pathway activation by bacterial components, an essential aspect of IBD development [76, 77]. These results are typical of pathogenic proinflammatory macrophages, known to release elevated amounts of TNF-α, IL-1β, IL-8, IL-6, CCL-2, and often associated with unresolving tissue injury [78, 79]. Such chronic inflammation obstructs proper wound healing and may promote a path for development of fibrosis [80, 81].

Although *PTEN* silencing is known to be associated with enhanced PI3K-AKT signaling [82], PTEN has also been reported to negatively regulate ERK activity [59, 68]. ERK1/2 activation was more prominent than AKT activation in *TET1*-deficient macrophages, suggesting that reduced *RBM38/PTEN* expression may preferentially contribute to elevated MAPK signaling. MAPK signaling promotes activation of AP-1 transcription factors through induction and phosphorylation of FOS and JUN family members, thereby enhancing their transcriptional activity at inflammatory gene loci. We found that loss of either *TET1* or *TET2* reduced 5hmC at AP-1-associated regulatory regions; however, *TET1* deficiency caused broader transcription factor motif dysregulation and gain of 5hmC at unique AP-1 (FOS/JUN) sites. TET1 is known to interact with transcriptional corepressors, and loss of TET1 recruitment may disrupt repressive chromatin complexes at specific loci, including bivalent promoters, resulting in both up and down regulation of gene expression [83–87]. Thus, loss of 5hmC in promoter regions cannot be uniformly interpreted as transcriptional activation or repression but rather reflects context-dependent alterations in transcription factor recruitment. *TET1* loss may also lead to gene activation and increased formation of 5hmC catalyzed by TET2 in the gene bodies of actively expressed genes [88–90]. Given that ERK enhances AP-1 function, and many inflammatory gene loci integrate NF-κB and AP-1 inputs [91–94], heightened MAPK signaling in *TET1*-deficient macrophages in addition to altered transcriptional accessibility by changes in 5hmC distribution likely contribute to differential outputs upon LPS/TLR4 signaling. These findings support a model in which *TET1* restrains inflammatory responses by limiting AP-1-dependent transcriptional amplification at MAPK-responsive inflammatory genes.

In response to LPS stimulation, our macrophage cell line models and patient samples showed enhanced production of chemokines such as CCL-2 and CCL-8, which are typically involved in leukocyte recruitment (i.e. neutrophils) and tissue inflammation. CCL-8 has also been described as an inflammatory biomarker of infection with human immunodeficiency virus, tuberculosis, in graft-versus-host disease, and in idiopathic pulmonary fibrosis [95]. Pleural effusion analysis from patients with tuberculosis also showed CCL-8 protein release occurred in a TLR2-dependent manner [96]. TLR2-dependent signaling can engage both PI3K-AKT and MAPK pathways and robustly activates ERK signaling. CCL-8 itself can activate ERK through CCR-mediated signaling, raising the possibility of a feed-forward MAPK amplification loop. Given that* TET1* loss specifically upregulates TLR2 surface expression in macrophages, it seems plausible that while *TET1* or *TET2* loss increases TNF-α pro-inflammatory cytokine secretion in response to LPS stimulation, *TET1* loss specifically amplifies chemokine signaling, potentially driven by enhanced MAPK signaling coupled with NF-κB activation. Regulation of NF-κB signaling and TET1 activity could also be bi-directional given that NF-κB/p65 binding to the promoter of *TET1* downregulates its expression [97] and we show that pre-emptive loss of *TET1* can elevate TNF-α production and chemokine expression via NF-κB activation that in turn would silence *TET1* in Crohn’s Disease and contribute to unresolved tissue inflammation.

Collectively, our data provides a novel rationale for assessing the efficacy of specific inhibitors targeting p38, ERK and NF-κB activity in the modulation of dysregulated chemokine signaling in Crohn’s Disease. While type I IFNs are essential for pathogen defense, their chronic overproduction is implicated in tissue damage, autoimmunity, and impaired resolution of inflammation [98]. Enhanced IFN gene expression signatures upon *TET1* loss suggests that TET1 may be required to restrain antiviral and inflammatory gene expression in macrophages and epithelia tissue, and its absence leads to a hyperresponsive state. Our data therefore highlights TET1 activity as a critical checkpoint in inflammation. Future studies should investigate whether restoring *TET1* expression or mimicking its activity can rescue *PTEN/RBM38* levels, reduce TLR2 upregulation, and reverse aberrant ERK activation to re-establish normal chemokine and IFN signaling. Implementing DNA hypomethylating agents and NF-κB inhibitors may also reverse *TET1* gene silencing and restore normal expression levels, offering a novel therapeutic strategy for the treatment of inflammatory bowel disease and other disorders characterized by immune suppression or chronic inflammation.

## Supplementary Information

Below is the link to the electronic supplementary material.

Supplementary Material 1

## Data Availability

Lead Contact: Resource and reagent requests should be directed to Luisa Cimmino ( luisa.cimmino@med.miami.edu ). Materials: This study generated new *TET1* and *TET2* knockout THP1 cell lines. These are available from the lead contact upon reasonable request and completion of a material transfer agreement (MTA). Data and code: This paper does not report original code. Any additional information required to reanalyze the data reported in this paper is available from the lead contact upon request.
