# Supplementary material for: TET1 deficiency amplifies macrophage inflammatory signaling associated with Crohn’s disease
Source: Inflamm Res. 2026 Mar 27;75(1):72. doi: 10.1007/s00011-026-02228-3 (PMC13031220; doi:10.1007/s00011-026-02228-3)

Supplementary Information

TET1 deficiency amplifies macrophage inflammatory signaling associated with Crohn's Disease.

Rocio K. Perez[1], Reeba Paul[2], Parveen Kumar[2], Alp Tutkun[2], Deborah Webb[1], Shea McGorty[1], Thomas Wieckowski[1], Yoon Sing Yap[3,4], Tiffany E. Leesang[3,4], Panayiotis I. Vlantis[3,4], Aristeidis G. Telonis[3,4], Christine Hajdin[2], Jim King[1], Gerald Nabozny[1], Luisa Cimmino[3,4*].

[1] *Immunology and Respiratory Department, Boehringer Ingelheim Pharmaceuticals, Inc, Ridgefield, Connecticut, USA.*

[2]*Computational Biology and Digital Sciences Department, Boehringer Ingelheim Pharmaceuticals, Inc, Ridgefield, Connecticut, USA.*

[3] *Department of Biochemistry and Molecular Biology, University of Miami, Miller School of Medicine, Miami, Florida, USA.*

[4] *Sylvester Comprehensive Cancer Center, Miami, Florida, USA.*

Supplementary Figures

Supplementary Figure 1: Generation of *TET1* and *TET2* CRISPR KO THP1 cell lines and analysis of surface marker expression, viability and growth. a) Sanger sequencing analysis of KO clones and **b)** taqman verification of CRISPR KO cell lines (upper panel) and 5hmC/5mC ELISA of genomic DNA (lower panels). **c)** Representative gating of equal distribution between unlabeled THP1 WT, CellTrace Violet labeled *TET1* KO, and CellTrace Far Red labeled *TET2* KO cells used for LEGENDScreen^{TM} analysis. **d)** Analysis of corresponding isotype controls across each cell genotype. **e)** Total mean fluorescence intensity (MFI) summary of surface marker expression on WT, *TET1* KO and *TET2* KO THP1 macrophages. **f)** Monocyte and macrophage (M0) cell viability, **g**) macrophage yield, and **h)** proliferation of THP1 monocytes assessed by CellTrace staining.

Supplementary Figure 2: Analysis of DNA methylation reprogramming in LPS stimulated *WT*, *TET1 KO* and *TET2 KO* THP1 macrophages. a) Fold-change in 5hmC and 5mC levels in *TET1 KO* and *TET2 KO* THP1 macrophages vs WT when stimulated with LPS. **b)** Volcano plots of total differential 5hmCs (DhmCs) measured by CpG-enriched EM-seq analysis (FDR <0.05, methylation differences >10%). **c)** Overlap of genes containing DhmCs that are gained or lost upon *TET1* or *TET2* KO.

Supplementary Figure 3: Transcriptional analysis of canonical and non-canonical LPS/TLR4 signaling genes in THP1 macrophages. Expression of genes associated with **a)** canonical and **b)** non-canonical LPS/TLR4 signaling. **c)** Percent of phospho-AKT and phospho-IRF3 compared to total protein levels quantified by flow cytometry in *WT*, *TET1* and *TET2* KO THP1 macrophages upon LPS stimulation.

Supplementary Figure 1 - Related to Figure 2

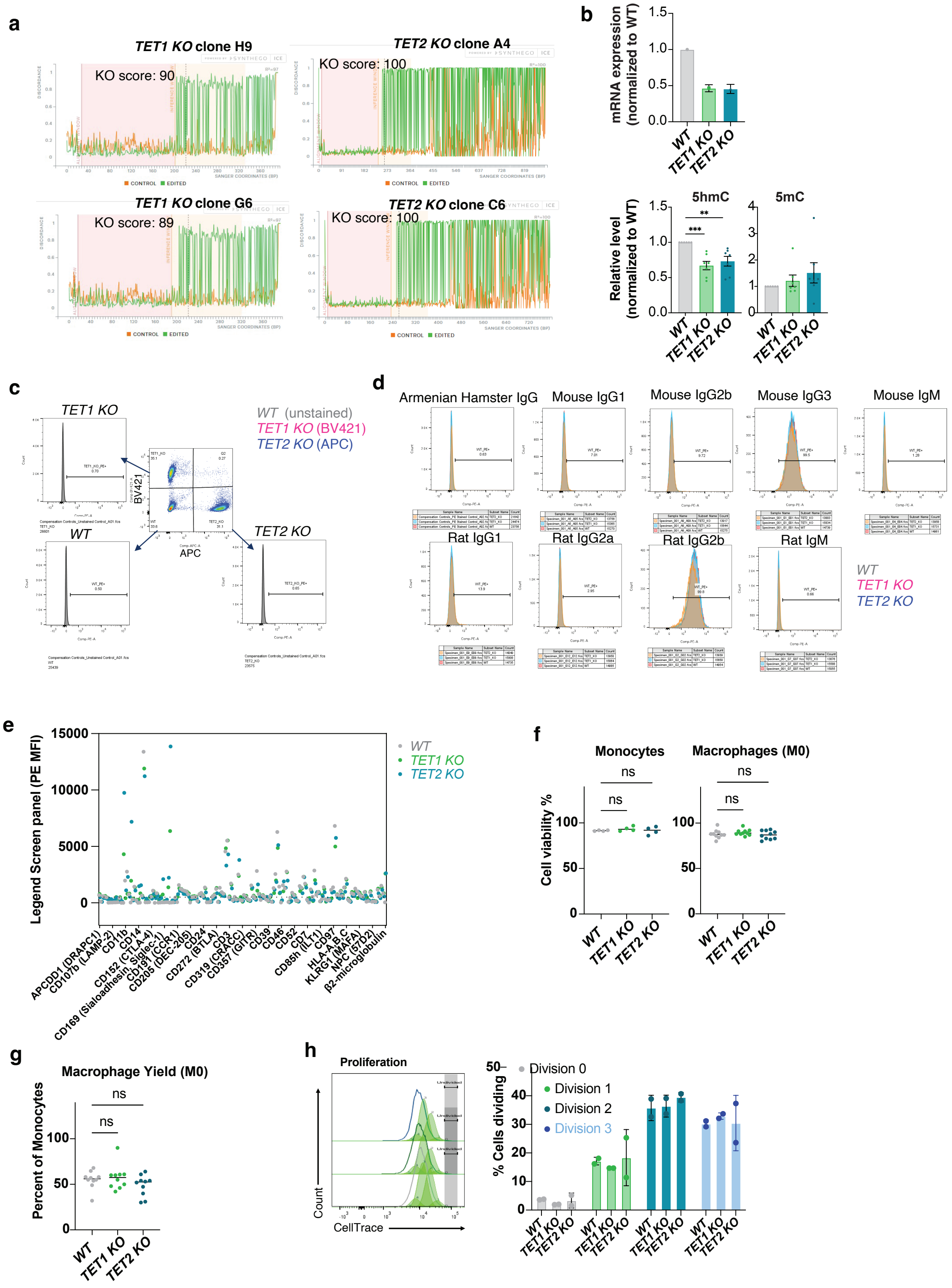

Supplementary Figure 2 - related to Figure 5

a

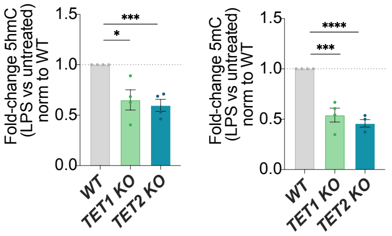

b

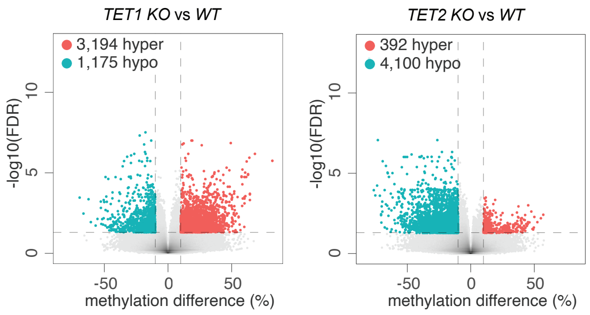

c

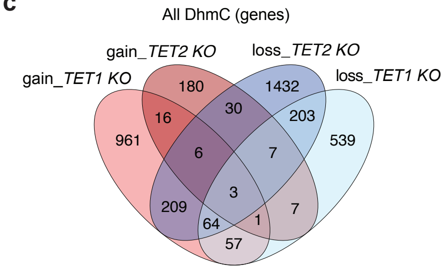

Supplementary Figure 3 - Related to Figure 6

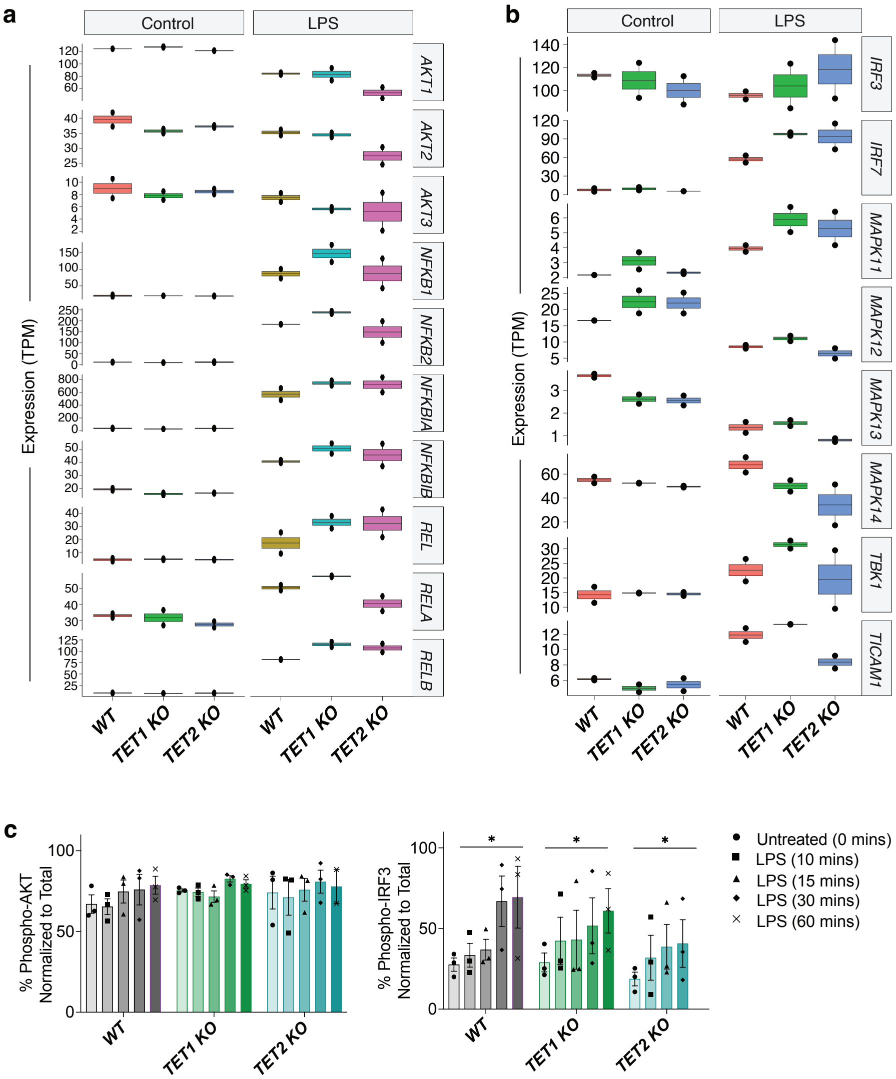

Supplement: Supplementary file 1 — Supplementary Material 1 [file 11_2026_2228_MOESM1_ESM.pdf]
